# The modulatory effect of evaluative valence on fear generalization in social anxiety: an SSVEP study

**DOI:** 10.3389/fnhum.2025.1586543

**Published:** 2025-09-16

**Authors:** Huoyin Zhang, Binyu Peng, Zihao Peng, Yi Lei

**Affiliations:** ^1^School of Psychology, Shenzhen University, Shenzhen, China; ^2^Institute of Brain and Psychological Science, Sichuan Normal University, Chengdu, China; ^3^College of Psychology, Sichuan Normal University, Chengdu, China; ^4^College of Education and Psychology, Southwest Minzu University, Chengdu, China

**Keywords:** social anxiety, fear generalization, evaluation valence, steady-state visual evoked potential, US expectancy ratings

## Abstract

**Background:**

Social anxiety is characterized by excessive sensitivity and concern about social evaluation. While previous research has demonstrated attentional bias and fear generalization in socially anxious individuals, the neural mechanisms by which different evaluative valences modulate this process remain unclear.

**Method:**

This study employed a fear generalization paradigm combined with steady-state visual evoked potentials (SSVEP). Fifty-one college students were recruited and divided into high (*n* = 25) and low (*n* = 26) social anxiety groups. A face-evaluation paradigm was used to record both behavioral and electroencephalographic (EEG) responses during fear generalization.

**Results:**

At the behavioral level, the high social anxiety group showed a broader fear generalization gradient. Both groups demonstrated higher unconditioned stimulus (US) expectancy ratings under negative versus positive evaluation conditions. This effect manifested differently between groups: in the high social anxiety group, it was specific to the conditioned stimulus (CS+) and generalization stimulus 4 (GS4), whereas in the low social anxiety group, it was observed for generalization stimulus 1 (GS1). At the neural level, SSVEP results revealed enhanced visual cortical activation (Oz, PO8) in the high social anxiety group across all stimuli. The PO7 electrode specifically reflected a differential modulation by evaluative valence between the groups; this adaptive modulation was evident in the low social anxiety group but absent in the high social anxiety group.

**Conclusion:**

This study reveals that social anxiety is characterized by a sustained state of early visual hypervigilance. Critically, we provide neurophysiological evidence that a core deficit underlying this condition is an impaired ability to utilize positive evaluation to down-regulate this hypervigilance. These results redefine our understanding of the cognitive-neural mechanisms of social anxiety, shifting the focus toward deficits in the neural processing of positive social information, and suggest that interventions should aim to restore the adaptive processing of positive social feedback.

## 1 Introduction

Consider a common scenario: During a conference presentation, a graduate student receives criticism about unclear research logic from an unfamiliar expert. Subsequently, this negative evaluation experience generalizes beyond the specific expert to others with similar appearances. This phenomenon of fear spreading from a specific negative experience to broader social contexts exemplifies what researchers term “fear generalization”. This generalization process, fundamentally rooted in learning theory and neural plasticity, represents a crucial mechanism in the development and maintenance of social anxiety tendencies. With the increasing pressure of academic evaluation and social media exposure in contemporary society, understanding such generalization processes becomes particularly relevant for mental health in academic and professional settings. Research indicates this phenomenon is especially prevalent among individuals with elevated social anxiety, and while adaptive fear learning helps individuals navigate social threats, excessive generalization can significantly impact academic performance and career development (Russell and Shaw, 2009).

Fear generalization is a crucial phenomenon in conditional fear learning, referring to the extension of fear responses from a conditioned stimulus (CS+, such as a sound paired with electric shock) to similar generalization stimuli (GS, such as similar tones) (Lissek et al., 2014). Recent systematic reviews and meta-analyses indicate that this fear generalization is a common phenomenon in pathological anxiety and positively correlates with anxiety severity (Fraunfelter et al., 2022). While research has begun to disentangle the influence of broad factors like threat context (Aslanidou et al., 2025) and uncertainty (Aslanidou et al., 2024) on this process, the specific role of social-evaluative valence—a key driver of social anxiety—in modulating fear generalization remains a critical, under-explored area. Our study aims to fill this gap by directly manipulating evaluative valence within a generalization paradigm. Experimental studies have found that anxious individuals demonstrate broader generalization gradients in fear generalization tasks, maintaining elevated fear responses even to GS with lower similarity to CS+. Researchers have validated this phenomenon through various experimental paradigms, including sound-shock pairing (Lissek et al., 2010) and face-sound pairing (Haddad et al., 2013), consistently showing excessive generalization characteristics in anxious individuals. This generalization response manifests not only in subjective reports and behavioral levels but also shows consistent findings in physiological indicators (such as skin conductance response).

In social anxiety, fear generalization holds particular clinical significance. Longitudinal studies have found that this generalization tendency can predict the developmental trajectory of social anxiety symptoms (Wong and Rapee, 2016) and the degree of social function impairment (Stein and Stein, 2008). Experimental research further reveals that compared to individuals with low anxiety, those with high social anxiety demonstrate steeper generalization curves in social situation generalization tasks, maintaining elevated anxiety responses even to scenarios with lower similarity to the original fear context (Ahrens et al., 2016). This excessive generalization characteristic is closely related to the core symptom of social anxiety: persistent worry about social evaluation. This persistent social evaluation concern may further exacerbate the degree of fear generalization through cognitive processing biases such as attentional bias (Dunsmoor and Paz, 2015), forming a vicious cycle.

The core characteristic of social anxiety is hypersensitivity to social evaluation. Behavioral research indicates that socially anxious individuals show significantly increased attention allocation when facing threatening faces (Bantin et al., 2016). This bidirectional evaluation fear aligns with the bivalent fear of evaluation model (BFOE), suggesting that social anxiety involves not only fear of negative evaluation (FNE) but also fear of positive evaluation (FPE). This bivalent nature of evaluation fears is supported by substantial evidence. Systematic reviews and meta-analyses have established robust links between both FNE and FPE and social anxiety (Cook et al., 2022; Fredrick and Luebbe, 2020), with longitudinal research confirming that these fears prospectively predict the development of social anxiety symptoms in adolescents (Fraunfelter et al., 2022). The BFOE model continues to be a vibrant area of research, with ongoing efforts to integrate its components (Hofmann, 2025) and delineate the shared and distinct mechanisms of negative and positive evaluation fears (Gao et al., 2025). Recent research has found that this evaluation sensitivity is closely related to specific cognitive processing biases, including attentional bias to social threat information (Mogg and Bradley, 2016), negative interpretation of ambiguous social cues (Hirsch and Clark, 2004), and selective recall of negative social memories (Mellings and Alden, 2000). More importantly, these cognitive biases, such as biased information-seeking and integration (Thyagaraj et al., 2025), may promote the maintenance and development of social anxiety by broadening the scope of perceived threats, thereby exacerbating the fear generalization process (Clark and Wells, 1995; Heimberg et al., 2014).

However, current research on fear generalization in social anxiety has several important limitations. First, existing studies have primarily focused on the impact of negative evaluation, with insufficient exploration of the potential modulatory role of positive evaluation. Although research has found that positive evaluation can also trigger fear responses in socially anxious individuals, the mechanism by which it influences the fear generalization process remains unclear. Second, existing studies largely rely on behavioral indicators and subjective reports, which make it difficult to capture dynamic changes in attention resource allocation during the fear generalization process. Third, there is a lack of systematic investigation into the neural mechanisms of how evaluation valence modulates fear generalization, particularly regarding neurophysiological evidence at the attention processing level.

Steady-state visual evoked potentials (SSVEPs) are oscillatory brain responses elicited when visual stimuli flicker at a constant frequency, typically observable as enhanced power at the driving frequency (e.g., 12 Hz) in posterior (occipital) electroencephalographic (EEG) electrodes (Norcia et al., 2015; Regan, 1989; Wieser et al., 2016). SSVEPs are characterized by their high signal-to-noise ratio (SNR) and precise frequency-domain definition, allowing researchers to reliably quantify sustained neural engagement with visual input over time (Wang and Yuan, 2021; Wieser et al., 2016). Functionally, SSVEP amplitude is enhanced when participants attend to, or process emotionally salient or threatening stimuli, reflecting increased allocation of attentional and perceptual resources in the visual cortex (Stegmann et al., 2023; Wang and Yuan, 2021; Wieser and Keil, 2014). For example, studies have shown that SSVEPs are amplified for emotionally arousing scenes or dynamic facial expressions compared to neutral or static stimuli, indicating heightened sustained processing of motivationally relevant information (Wang and Yuan, 2021; Wieser and Keil, 2014). Thus, SSVEPs provide a robust and temporally precise index of sustained visuocortical engagement, sensitive to both bottom-up sensory properties and top-down modulatory factors such as attention and emotion.

Compared to traditional behavioral measurements and subjective reports, SSVEP has significant methodological advantages: First, it provides extremely high temporal resolution, enabling real-time tracking of dynamic attention resource allocation processes (Norcia et al., 2015); second, SSVEP signals have a high SNR, making attention process measurements more reliable; third, as an objective indicator, SSVEP is less influenced by cognitive control. Indeed, SSVEP has become a powerful research tool in social affective neuroscience (Wieser et al., 2016), capable of tracking the neural processing of dynamic facial expressions (Wang and Yuan, 2021). Recent research has revealed dynamic characteristics of visual cortical responses in socially anxious individuals when facing social threats, potentially manifesting as hypervigilance or avoidance (McTeague et al., 2018). Particularly noteworthy is a recent finding suggesting that social aversive generalization learning sharpens the tuning of visual cortical neurons to facial features. Specifically, this learning process enhances neural selectivity for threat-related faces while suppressing responses to highly similar faces, a pattern indicative of lateral inhibition that may form the neural basis of attentional bias in socially anxious individuals (Stegmann et al., 2020). Building on this, recent work has begun to map the sustained neurophysiological dynamics of social fear generalization using techniques like magnetoencephalography (MEG) to track responses over time (Pouliot et al., 2025).

However, these studies have not systematically manipulated the valence of the social feedback, which is a core component of social anxiety. Our study directly addresses this gap by using SSVEP to investigate how both positive and negative evaluations distinctly shape the neural correlates of generalization, highlighting the timeliness and importance of our approach.

Based on this research background, the present study aims to employ a fear generalization paradigm combined with SSVEP technology to investigate the modulatory effect of evaluation valence on attention generalization characteristics in socially anxious individuals. We hypothesize that: (1) The high social anxiety group will show stronger SSVEP responses to CS+ and similar GS compared to the low social anxiety group; (2) Evaluation valence will modulate this attention generalization effect, with more pronounced generalization effects under negative evaluation conditions, while positive evaluation may narrow the generalization range. These findings will not only provide new neurophysiological evidence for attention processing mechanisms in social anxiety but also hold significant clinical application value.

## 2 Materials and methods

### 2.1 Participants

A priori efficacy analyses were conducted in this study using G*Power 3.1. A mixed repeated-measures ANOVA design for 2 (group: high social anxiety, low social anxiety) × 2 (evaluation type: positive, negative) × 6 (stimulus type: CS−, GS1, GS2, GS3, GS4, CS+) was used to set an expected medium effect size of f = 0.25, α = 0.05, power = 0.95, and a minimum sample size of 18 subjects.

Following these sample size requirements, we employed a multi-stage screening process. Initially, the Liebowitz Social Anxiety Scale (LSAS; He and Zhang, 2004; Liebowitz, 1987) was distributed through the Brain Island online platform (Chen et al., 2023) and social networking platforms. Of 1,858 questionnaires distributed, 1,445 valid responses were received (78% response rate). Participants were divided into high and low social anxiety groups based on the LSAS total score median (62 points), with the high social anxiety group scoring ≥62 and the low social anxiety group scoring <62 (Glass et al., 1982). Subsequently, eligible participants were invited to participate in the experiment. Fifty-five university students were initially recruited, with four excluded due to excessive EEG artifacts, resulting in a final sample of 51 participants. The high social anxiety group comprised 25 participants (21 females; mean age 19.92 ± 1.22 years), and the low social anxiety group included 26 participants (18 females; mean age 19.58 ± 1.39 years) (see Table 1). All participants were right-handed with normal or corrected-to-normal vision, no history of psychiatric or major physical illness, and no prior participation in similar experiments.

**TABLE 1:** Demographic characteristics and clinical measures by group (M ± SD).

Variable	High social anxiety (*n* = 25)	Low social anxiety (*n* = 26)	*t*_49_/ χ ^2^	*p*	Cohen’s *d*
Gender (male/female)	4/21	8/18	1.55	0.21	
Age (years)	19.92 ± 1.22	19.58 ± 1.39	6.18	0.40	0.32
LSAS (total score)	80.68 ± 12.96	28.85 ± 11.48	15.133	<0.001	4.73
LSAS - fear/anxiety	41.88 ± 8.32	14.19 ± 6.35	13.394	<0.001	3.75
LSAS - avoidance	39.84 ± 10.45	14.65 ± 8.70	9.365	<0.001	2.62

LSAS, Liebowitz Social Anxiety Scale (range 0–144; Fear subscale range 0–72; Avoidance subscale range 0–72). Independent samples *t*-tests were used for continuous variables; chi-square test was used for gender comparison. Cohen’s d: 0.2–0.5 small effect, 0.5–0.8 medium effect, ≥ 0.8 large effect.

All participants provided written informed consent and received compensation upon completion. The study was approved by the Ethics Committee of the Institute of Brain and Psychological Sciences at Sichuan Normal University (approval number SCNU-211120, approved November 20, 2021) and conducted in accordance with the Declaration of Helsinki (World Medical Association, 2013).

### 2.2 Experimental materials

Conditioned stimuli (CS) were selected from three female neutral expression faces in the NimStim face expression database (Tottenham et al., 2009), chosen for its established reliability and validity in emotion research (Paulus and Wentura, 2016). To hold stimulus gender constant and thereby eliminate it as a potential confounding variable, only female faces were used. Following Onat and Büchel (2015) procedure, the conditioned threat stimulus (CS+) faces were gradually morphed into the conditioned safety stimulus (CS−) faces in 20% increments, creating four gradient levels (GS1, GS2, GS3, GS4). The 20% increment was selected to produce perceptually continuous generalization gradients (Stegmann et al., 2020). All facial stimuli (CS and GS) were converted to grayscale to standardize presentation.

Unconditioned stimuli (US) comprised 12 evaluative statements selected from the Chinese Personality Trait Adjective Pool (Huang and Zhang, 1992), including high-arousal positive and negative evaluations. Positive evaluations included: “You are interesting,” “You are reliable,” “You are humorous,” “You are capable,” “You are polite,” and “You are sincere.” Negative evaluations included: “You are selfish,” “You are hypocritical,” “You are cunning,” “You are shameless,” “You are opportunistic,” and “You are mean.”

### 2.3 Experimental procedure

**FIGURE 1:**
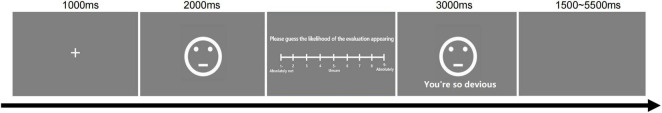
Schematic diagram of the experimental process.

To create an authentic social context, participants were asked to provide a color ID photo before the experiment and were informed that it would be used in an online interaction task where they would evaluate others’ photos while their own photos would be evaluated by others (in reality, there were no other participants, and photos were from the database). The formal experiment consisted of habituation, acquisition, and generalization phases (Figure 1).

During the habituation phase, CS+Positive, CS+Negative, and CS– were each presented three times in pseudo-random order (without repetition between trials). No CS was paired with US in this phase, and data were not recorded. This phase aimed to familiarize participants with the experimental procedure. In the acquisition phase, CS+Positive, CS+Negative, and CS– were each presented eight times across 24 trials in pseudo-random order. CS+Positive and CS+Negative were paired with US Positive and US Negative, respectively at a 75% probability. On the remaining 25% of trials, these CS+ stimuli were presented without an accompanying US. CS– was never paired with any US. The assignment of CS+Positive and CS+Negative was counterbalanced across participants. The generalization phase comprised four blocks of 22 trials each. In each block, CS+Positive, CS+Negative, and CS– were presented twice, and each GS was also presented twice. To prevent memory extinction, one CS+ was paired with its corresponding US in each block. All stimuli were presented in pseudo-random order to ensure temporal distribution of stimulus types.

The experimental procedure was programmed using E-Prime 3.0 (Psychological Software Tools, Inc., Pittsburgh, PA, USA). Each trial began with a 1000 ms fixation point, followed by a 2000 ms presentation of CS or GS. Participants then rated US expectancy on a 1–9 scale, with higher numbers indicating greater probability. The 1–9 rating scale was chosen for its ability to capture subtle differences in participants’ responses, particularly when assessing subjective expectations of positive or negative evaluations. Following the rating, a face with an evaluation or a neutral face was presented for 3000 ms. Inter-trial intervals ranged from 9000 to 12000 ms. The entire experiment was conducted in a quiet laboratory and lasted 60–70 min.

### 2.4 EEG preprocessing and analysis

Electroencephalographic data were recorded using a 64-channel Ag/AgCl electrode cap (EEGosports, BP Inc.), with electrode placement following the international 10–20 system. Data were recorded at a sampling rate of 500 Hz with DC compensation, and all electrode impedances were maintained below 10 kΩ. EEG signal preprocessing was performed offline using EEGLAB v14.1.1b (Delorme and Makeig, 2004) in MATLAB (R2021b). Continuous EEG data were then bandpass-filtered from 0.1 to 40 Hz and a 50 Hz notch filter was applied to remove power line noise. Trial data segments from −500 ms to +2000 ms were extracted, using the stimulus onset as the reference. Subsequently, independent component analysis (ICA) based on the Infomax algorithm was applied to the segmented data to further correct artifacts. Specifically, ICA is used to identify and remove components associated with eye movements, blinking, electromyography (EMG) activity, electrocardiography (ECG) artifacts, and non-physiological noise. Interference components are manually identified based on morphological criteria described by Chaumon et al. (2015). Finally, time segments with amplitudes exceeding ± 100 μV at any electrode are automatically rejected (Chaumon et al., 2015).

Following preprocessing, data analysis proceeded as follows: first, peripheral electrodes (IO, TP9, TP10) were removed, and the data were converted to an average reference to enhance spatial specificity, followed by current source density (CSD) transformation to improve spatial resolution (Kayser and Tenke, 2006). Second, a fast Fourier transform (FFT) analysis is performed on a 500–2000 ms analysis window to extract the 12 Hz SSVEP signal. This frequency is selected based on its superior SNR characteristics in visual evoked responses (Di Russo et al., 2007). The SNR is calculated by dividing the power at 12 Hz by the average power of the six adjacent frequency bands (excluding the two immediately adjacent bands) to ensure the reliability of the measurement (Regan, 1989). This calculation method has been validated in recent studies (Barry-Anwar et al., 2018; Stegmann et al., 2020, 2023), and the SNR at 12 Hz is defined as follows:


$${\text{SNR}}_{12\,\text{Hz}}=\frac{P_{12\,\text{Hz}}}{\frac{1}{6}\,\sum_{i=1}^{6}\times P_{a\,d\,j,i}}$$

Based on previous studies (Norcia et al., 2015; Rossion et al., 2012), the occipital electrodes Oz, PO7, and PO8 were selected as analysis sites.

### 2.5 Statistical methods

Statistical analyses were performed using SPSS 27.0 (IBM Corp., Armonk, NY, USA). Dependent variables were US expectancy ratings and 12 Hz SNR values. Acquisition phase data were analyzed using 2 (Group: high/low social anxiety, between-subjects) × 3 (Stimulus type: CS+positive/CS–/CS+negative, within-subjects) repeated measures ANOVA. Generalization phase data were analyzed using 2 (Group: high/low social anxiety, between-subjects) × 2 (Evaluation valence: positive/negative, within-subjects) × 6 (Stimulus type: CS–/GS1/GS2/GS3/GS4/CS+, within-subjects) repeated measures ANOVA. Statistical significance was set at *p* < 0.05, with Greenhouse-Geisser correction applied when sphericity was violated. Where necessary, Bonferroni-corrected simple contrasts were computed as *post-hoc* tests. Effect sizes were reported as η_p_^2^ for ANOVA results and Cohen’s d for *post-hoc* comparisons.

## 3 Results

### 3.1 Acquisition results

**FIGURE 2:**
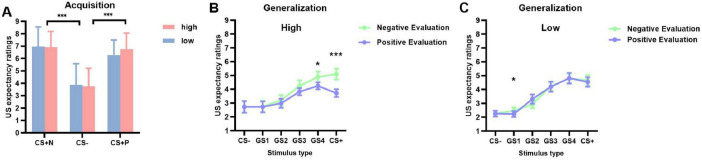
US expectancy ratings during acquisition and generalization phases (M ± SD). **(A)** Acquisition phase ratings for CS+Negative, CS–, and CS+Positive stimuli: red bars represent high social anxiety group and blue bars represent low social anxiety group. **(B,C)** Generalization phase gradient responses from CS– through GS1-4 to CS+ in high anxiety group **(B)** and low anxiety group **(C)**: light green lines represent negative evaluations and light blue lines represent positive evaluations. *p < 0.05, ***p < 0.001.

A significant main effect of stimulus type was observed [*F*(2, 98) = 70.713, *p* < 0.001, η_p_^2^ = 0.591]. This indicates significant differences in US expectancy ratings across different stimulus types (CS+ positive, CS+ negative, and CS–). Subsequent pairwise comparisons revealed no significant difference between CS+ positive (*M* = 6.937, SD = 0.202) and CS+ negative (*M* = 6.520, SD = 0.175, *p* = 0.211), but both were significantly higher than CS– (*M* = 3.810, SD = 0.221, *ps* < 0.001). No other effects reached significance (*ps* > 0.05) (Figure 2A).

### 3.2 Generalization results

#### 3.2.1 US expectancy ratings

Analysis revealed a significant main effect of stimulus type [*F*(5, 245) = 63.945, *p* < 0.001, η_p_^2^ = 0.566]. US expectancy ratings showed an overall decreasing trend from CS+ (*M* = 4.506, SD = 0.217) to CS– (*M* = 2.490, SD = 0.234). *Post-hoc* pairwise comparisons showed no significant difference between CS+ (*M* = 4.506, SD = 0.217) and GS4 (*M* = 4.687, SD = 0.222, *p* = 0.293). CS+ was significantly higher than GS3 (*M* = 4.129, SD = 0.208, *p* = 0.006), GS2 (*M* = 3.104, SD = 0.211, *p* < 0.001), GS1 (*M* = 2.539, SD = 0.229, *p* < 0.001), and CS– (*M* = 2.490, SD = 0.234, *p* < 0.001). GS4 was also significantly higher than GS3, GS2, GS1, and CS– (*ps* < 0.001). GS3 was significantly higher than GS2, GS1, and CS– (*ps* < 0.001). GS2 was significantly higher than GS1 (*p* < 0.001) and CS– (*p* = 0.003), while no significant difference was found between GS1 and CS– (*p* = 1.000). A significant interaction between stimulus type and evaluation valence emerged [*F*(5, 245) = 4.332, *p* < 0.001, η_p_^2^ = 0.081]. No other main effects or two-way interactions were significant (*ps* > 0.05).

A significant three-way interaction among stimulus type, evaluation valence, and group was found [*F*(5, 245) = 3.811, *p* = 0.022, η_p_^2^ = 0.076]. Simple effects analysis revealed that in the high social anxiety group, US expectancy ratings were significantly higher under negative evaluation conditions compared to positive evaluation conditions for CS+ and GS4 (*ps* < 0.05). Specifically, for CS+ (*M+negative* = 5.100, SD = 0.390; *M+positive* = 3.718, SD = 0.321) and GS4 (*M+negative* = 4.898, SD = 0.381; *M+positive* = 4.244, SD = 0.326) (Figure 2B). The low social anxiety group showed a significant difference only for GS1, in the same direction (*M+negative* = 2.468, SD = 0.341; *M+positive* = 2.227, SD = 0.310, *p* = 0.036) (Figure 2C). No significant differences were found between positive and negative evaluation conditions for GS2, GS1, and CS– in either group (*ps* > 0.05).

#### 3.2.2 SSVEP signal-to-noise ratio (SNR)

**FIGURE 3:**
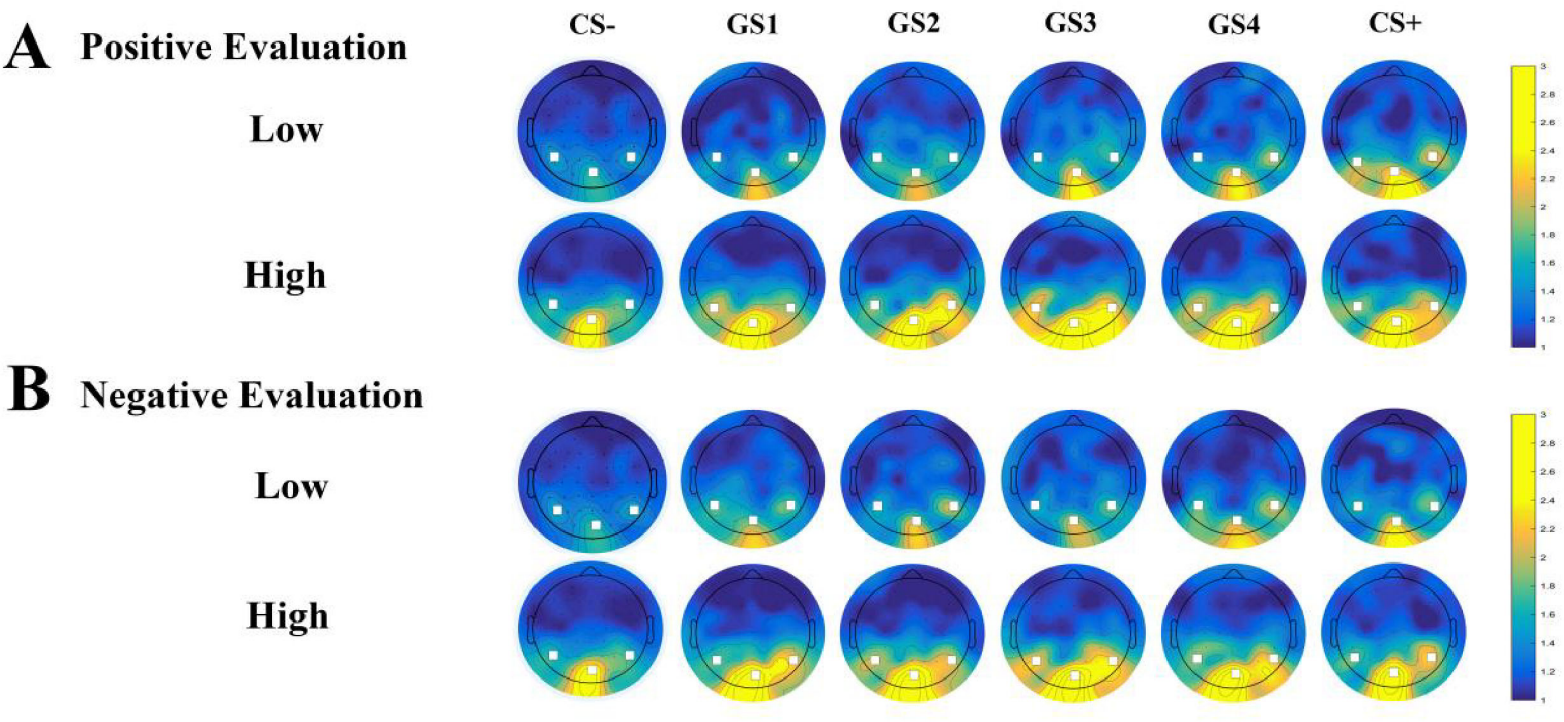
Topographical map of 12 Hz SSVEP signal-to-noise ratio (SNR). **(A)** SNR distribution topographical map under positive evaluation conditions. **(B)** SNR distribution topographical map under negative evaluation conditions. Color scale represents SNR values (range: 1–3). Warmer colors indicate higher SNR values.

**FIGURE 4:**
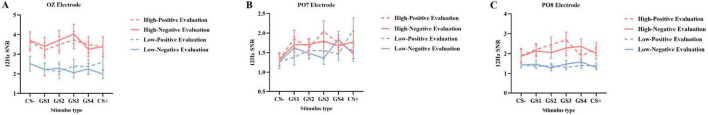
12 Hz SSVEP signal-to-noise ratio (SNR) during the generalization phase (M ± SD). Differences in SNR at Oz **(A)**, PO7 **(B)**, and PO8 **(C)** electrodes across different social anxiety levels: red represents the high social anxiety group, blue represents the low social anxiety group; dashed lines represent positive evaluations, solid lines represent negative evaluations.

Oz Electrode SNR (Figure 3): Results showed a significant main effect of group [*F*(1, 49) = 7.050, *p* = 0.011, η_p_^2^ = 0.126]. The high social anxiety group (*M* = 3.562, SD = 0.352) showed significantly higher SNR than the low social anxiety group (*M* = 2.254, SD = 0.345). A significant interaction between group and stimulus type was observed [*F*(5, 245) = 3.022, *p* = 0.011, η_p_^2^ = 0.058]. Simple effects analysis revealed higher SNR values in the high social anxiety group across all stimulus types. For GS4 through GS1, group differences reached statistical significance (GS4: *p* = 0.031; GS3: *p* = 0.015; GS2: *p* = 0.003; GS1: *p* = 0.029), with the high social anxiety group consistently showing higher SNR values. The most pronounced group difference was observed for CS– (high anxiety: *M* = 3.697, SD = 0.351; low anxiety: *M* = 1.991, SD = 0.344; *p* = 0.001). No other effects reached significance (*ps* > 0.05) (Figure 4A).

PO8 Electrode SNR (Figure 3): Results showed a significant main effect of group [*F*(1, 49) = 7.099, *p* = 0.010, η_p_^2^ = 0.127]. The high social anxiety group (*M* = 2.205, SD = 0.220) showed significantly higher SNR than the low social anxiety group (*M* = 1.385, SD = 0.215). No other effects reached significance (*ps* > 0.05) (Figure 4C).

**FIGURE 5:**
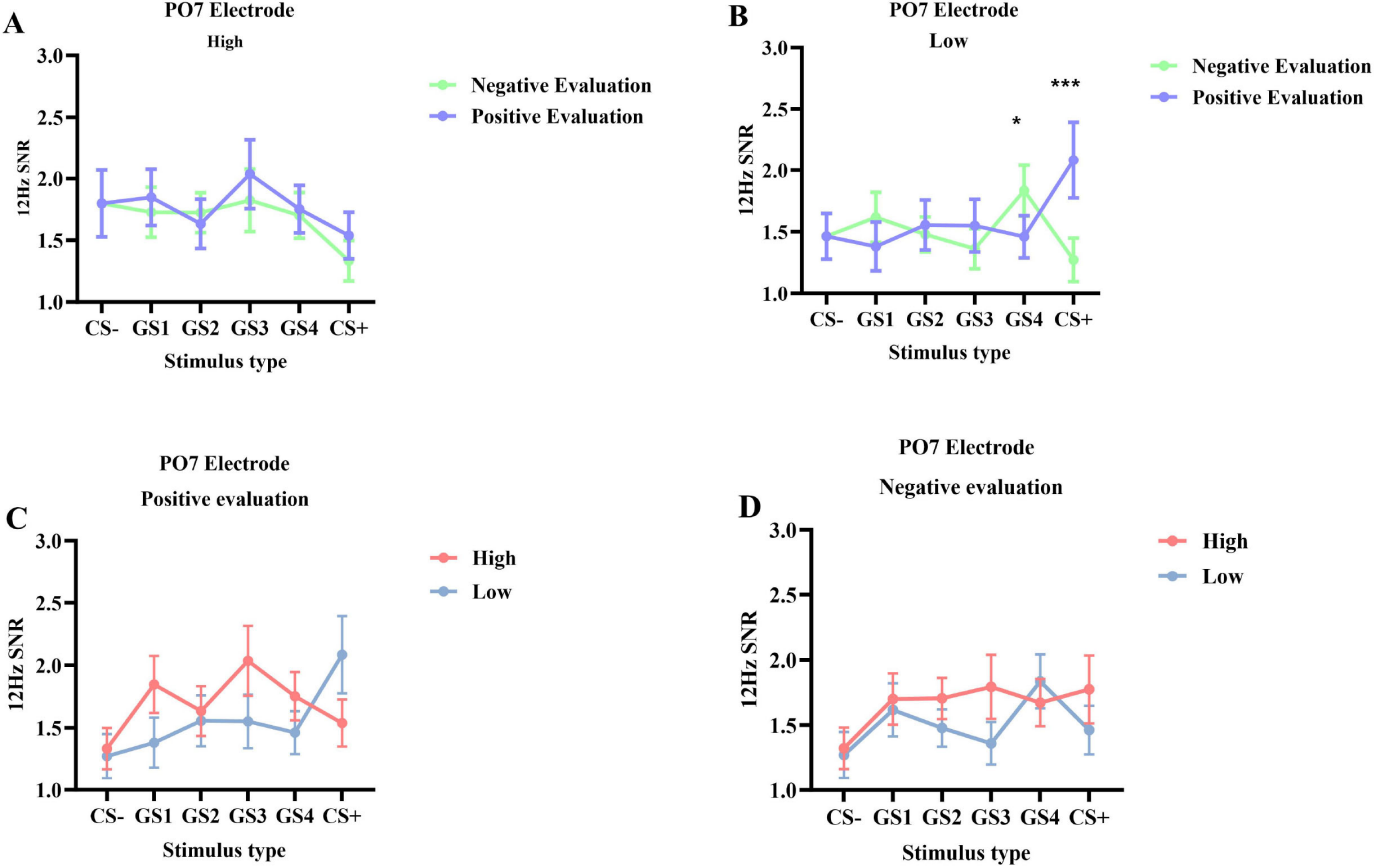
The 12 Hz SSVEP signal-to-noise ratio (SNR) at the PO7 electrode during the generalization phase (M ± SD). This figure illustrates the interaction between social anxiety group and evaluation condition from two perspectives. Panels **(A,B)** show the effect of evaluation type, comparing negative evaluation (light green line) versus positive evaluation (blue line) separately within the high **(A)** and low **(B)** social anxiety groups. Conversely, panels **(C,D)** show the effect of the anxiety group, comparing the high (red line) versus low (blue line) social anxiety groups separately under the positive evaluation condition **(C)** and the negative evaluation condition **(D)**. Asterisks indicate significant differences: *p < 0.05, ***p < 0.001.

PO7 Electrode SNR (Figure 3): Results showed a significant interaction between stimulus type and group [*F*(5, 245) = 2.762, *p* = 0.019, η_p_^2^ = 0.053], as well as a significant interaction between stimulus type and evaluation valence [*F*(5, 245) = 4.109, *p* = 0.001, η_p_^2^ = 0.077]. No other main effects or two-way interactions were significant (*ps* > 0.05). A significant three-way interaction among stimulus type, evaluation valence, and group was found [*F*(5, 245) = 2.505, *p* = 0.046, η_p_^2^ = 0.049]. Simple effects analysis showed that in the low social anxiety group, SNR under positive evaluation conditions (*M* = 2.085, SD = 0.256) was significantly higher than under negative evaluation conditions (*M* = 1.272, SD = 0.170, *p* < 0.001) for GS+ stimuli. For GS4 stimuli, SNR under positive evaluation conditions (*M* = 1.461, SD = 0.180) was significantly lower than under negative evaluation conditions (*M* = 1.835, SD = 0.196, *p* = 0.014) (Figures 4B, 5).

## 4 Discussion

This study employed a fear generalization paradigm combined with SSVEP technology to investigate the modulatory effect of evaluative valence on fear generalization characteristics in socially anxious individuals. The results supported our main hypotheses: the high social anxiety group demonstrated broader fear generalization characteristics, and this generalization pattern was significantly modulated by evaluative valence. This modulatory effect was evident at both behavioral and neurophysiological levels: behavioral data showed that both groups demonstrated higher US expectancy ratings under negative versus positive evaluation conditions, specifically for CS+ and GS4 in the high social anxiety group and for GS1 in the low social anxiety group. SSVEP data revealed the neural mechanisms of this effect, with enhanced visual cortical activation observed in the high social anxiety group at Oz and PO8 locations, and specific modulatory effects of evaluative valence observed at PO7. These multi-level experimental findings deepen our understanding of fear generalization mechanisms in social anxiety and provide neurophysiological evidence for the modulatory role of evaluative valence.

### 4.1 Behavioral and neural mechanisms of fear generalization

To better understand these findings, analysis at both behavioral and neural levels is necessary. Regarding behavioral characteristics of fear generalization, our results indicate that socially anxious individuals exhibit unique generalization patterns. First, acquisition phase results showed successful establishment of CS+-US associations, laying the foundation for subsequent generalization effects. In the generalization phase, we observed patterns partially consistent with but also different from Ahrens et al. (2016). While their study found enhanced US expectancy across all generalization stimuli in the high social anxiety group, our research, by introducing the modulatory role of evaluative valence, revealed a more complex picture: both groups showed higher US expectancy ratings under negative versus positive evaluation conditions, but this effect manifested in different generalization stimuli across groups - the high social anxiety group primarily showed this effect in stimuli similar to the threat stimulus (CS+ and GS4), while the low social anxiety group showed significant effects in stimuli similar to the safety stimulus (GS1).

This dissociation pattern has important theoretical implications: first, it indicates that the influence of evaluative valence on fear generalization is universal, not limited to highly socially anxious individuals; second, it reveals that high and low socially anxious individuals may have different threat-safety information processing patterns, with highly socially anxious individuals being more sensitive to threat-related information, while low socially anxious individuals also showed evaluative valence modulation in the processing of safety-related information.

The SSVEP technique provided new insights into the neural mechanisms of fear generalization. Results revealed distinct spatial distribution patterns: the Oz region reflected basic visual processing characteristics, while PO7 and PO8 demonstrated lateralization features in face processing. This spatial distribution pattern aligns with Rossion and Boremanse (2011) findings regarding neural network organization characteristics in face processing. Compared to existing studies primarily relying on behavioral indicators and subjective reports, SSVEP technology enabled us to observe enhanced visual cortical activation in these regions in the high social anxiety group. This finding is consistent with the notion that threat-related stimuli, such as fearful faces or contexts associated with threat, heighten cortical representation and gain preferential processing (Wieser and Keil, 2014).

Furthermore, our SSVEP results meaningfully complement Wieser et al. (2016) study using facial stimuli. Although both studies employed facial stimuli, important distinctions exist: Wieser et al. (2016) primarily examined processing differences for emotional faces (angry, happy, etc.), while we focused on generalization effects of neutral faces under different evaluative contexts. Our observation of enhanced visual cortical activation in the high social anxiety group at Oz and PO8 not only replicates Wieser and Keil (2014) findings in basic visual processing but, more importantly, demonstrates how this enhancement effect is modulated by evaluative context.

Particularly noteworthy is the three-way interaction observed at PO7, indicating that evaluative valence modulation occurs during early stages of face processing. This finding complements Stegmann et al. (2020) SSVEP research: while they found that social aversive learning affects neural responses to facial features in the visual cortex, our study further reveals the specific manifestation of this modulatory effect in evaluative contexts, particularly in differential patterns between individuals with varying levels of social anxiety. This pattern of impaired discrimination in high-anxious individuals also resonates with findings from Stegmann et al. (2019), who similarly observed that high trait-anxious individuals failed to differentiate between contextual threat cues in a conditioning paradigm. Our study extends this by demonstrating a similar discrimination deficit in a social evaluation context and linking it specifically to the valence of the evaluation. However, the stronger response to negative versus positive evaluation for GS4 in the low-anxiety group was an unexpected finding. We interpret this isolated result with caution, as it does not align with our main hypotheses and may represent statistical noise rather than a theoretically meaningful effect.

An intriguing finding emerged from the low social anxiety group, where there was an apparent dissociation between behavioral and neural findings. Behaviorally, the modulatory effect of valence was significant only for GS1, a stimulus near the safety end of the generalization spectrum. Neurally, however, the effect was uniquely prominent for the CS+, the threat-related stimulus. This apparent discrepancy is highly informative, likely reflecting the distinct psychological processes captured by each measure. The US expectancy rating is a conscious, cognitive evaluation of future probability, a process often considered part of a “high road” of emotional processing (Pessoa and Adolphs, 2010). In this context, the healthy brain may be most sensitive to subtle valence modulations at the boundary of safety (GS1). In contrast, the SSVEP response reflects a more automatic allocation of sustained attentional resources to the stimulus being processed (Norcia et al., 2015; Wieser and Keil, 2014). For the low-anxiety group, the CS+ signaling a positive outcome becomes the most motivationally salient cue, adaptively commanding the most neural resources. This highlights that fear generalization is not a monolithic process and underscores the importance of using multi-level measurements to disentangle explicit cognitive prediction from implicit attentional processing.

### 4.2 Modulatory effects of evaluative valence

This study revealed significant modulatory effects of evaluative valence on fear generalization, which has important implications for understanding the cognitive-neural mechanisms of social anxiety. These modulatory effects were evident not only at the behavioral level but also in neural activity. Our behavioral results showed that negative evaluation, compared to positive evaluation, elicited stronger US expectancy responses, with this effect manifesting in threat-related stimuli in the high social anxiety group and in safety-related stimuli in the low social anxiety group. These findings partially support Lissek et al. (2014) perspectives on generalization characteristics in anxious individuals while extending this theoretical framework by revealing group-specific modulatory effects of evaluative valence.

Perhaps the most critical finding of this study is the nature of the valence modulation at the PO7 electrode. This site, which is associated with left-hemisphere contributions to emotional face processing (Nagy et al., 2012), reveals a specific neural deficit in social anxiety. The low-anxiety group demonstrated an adaptive neural response by significantly enhancing attentional processing for positive CS+ cues, whereas the high-anxiety group failed to show any such differentiation. Their neural response at PO7, combined with their generally elevated activation at Oz and PO8, a pattern consistent with right-hemisphere dominance in face processing (Rossion et al., 2012), suggests that their visual cortex remains in a state of undifferentiated hypervigilance. This indicates that the core deficit is not merely an over-reaction to negative information, but a fundamental inability to utilize positive social cues to down-regulate this default state of hypervigilance. This specific neural mechanism provides a potential substrate for the BFOE model, which posits that socially anxious individuals also fear positive evaluation (Fredrick and Luebbe, 2020). Our data suggest this may stem from a failure, at the neural level, to process positive feedback in a manner that adaptively reduces this defensive vigilance. This neural inflexibility persists even when, at a behavioral level, they can explicitly report different expectancies, highlighting a profound dissociation between cognitive awareness and early, automatic neural processing in social anxiety.

The current study demonstrates that the modulatory effects of evaluative valence on fear generalization may begin at early stages of visual processing. Previous research has established the involvement of early visual cortical areas in fear generalization (Dunsmoor and Paz, 2015; Lissek et al., 2014), and indicated that individuals with social anxiety are particularly sensitive to evaluative cues (Morrison and Heimberg, 2013; Stein and Stein, 2008). This suggests that evaluation sensitivity in social anxiety may modulate subsequent fear generalization processes via early visual mechanisms.

### 4.3 Research implications and limitations

These findings have important implications for theoretical development and clinical practice. Our findings provide significant modifications to theories of cognitive-neural mechanisms in social anxiety. First, our results support and extend Fredrick and Luebbe (2020) BFOE model. The finding that evaluative valence manifests at early visual processing stages not only validates Peschard and Philippot (2016) theoretical framework but also indicates the need to incorporate perceptual processes into theoretical models of social anxiety. This directly supports McTeague et al. (2018) “bottom-up” processing model while prompting reconsideration of Heeren and McNally (2018) network model of social anxiety disorder. Our findings suggest that perceptual processes and evaluative valence are key nodes within the complex symptom network of social anxiety, and that early sensory processing deficits may interact with evaluative beliefs to sustain maladaptive fear generalization. Specifically, perceptual-level interventions could be added to Hofmann et al. (2012) attention training protocol. Second, the modulatory role of evaluative valence suggests that exposure therapy should systematically incorporate different types of evaluative contexts. This can be achieved by improving Clark and Wells (1995) cognitive restructuring techniques, such as designing exposure hierarchies specifically targeting different evaluation types.

However, this study has several main limitations. First, while we observed modulatory effects of evaluative valence, these modulatory mechanisms may be influenced by multiple factors. Previous research indicates that individual responses to social evaluation may depend on evaluation source (Blair et al., 2008), evaluation content (Weeks et al., 2010), and individual cognitive interpretation patterns (Rapee and Heimberg, 1997). For example, Heimberg et al. (2014) found that some socially anxious individuals might interpret positive evaluation as raising others’ expectations, thereby generating more anxiety. This complex cognitive-emotional interaction pattern was not fully explored in our study. Second, laboratory setting limitations may affect the ecological validity of research results. As Primack et al. (2017) pointed out, evaluations in modern social environments often have multi-source, dynamic, and interactive characteristics, especially in social media environments. This complexity is difficult to fully simulate under laboratory conditions. Freeman et al. (2017) research suggests that virtual reality technology may offer new possibilities for improving the ecological validity of social anxiety research. Third, a limitation is the gender composition of our sample. Our study included a predominantly female sample. While this reflects the higher prevalence of social anxiety in women (Stein and Stein, 2008), it restricts the generalizability of our conclusions. Given that our design exclusively used female faces, the observed neural and behavioral patterns may be specific to how females with social anxiety process evaluations delivered by other females. Future research should therefore recruit a more gender-balanced sample to test whether these findings extend to males and to explore potential interactions between participant and stimulus gender.

Based on these findings and limitations, future research could proceed in several directions: First, systematic investigation of evaluative valence mechanisms is needed, including the influence of evaluation source (authority vs. peers), evaluation content (ability vs. traits), and individual characteristics (such as evaluation sensitivity). For example, combining virtual reality technology (Carl et al., 2019) and multimodal measurements (Lazarov et al., 2019) may help better understand the mechanisms of evaluative valence in real social situations. Furthermore, interventions could be developed to target these early perceptual processes. Future work might explore the efficacy of perceptual training paradigms, perhaps using novel dynamic stimuli, such as the flickering videos employed by Stegmann et al. (2023) to successfully measure sustained attention in different threat contexts. This technique shows promise for both assessing and potentially modifying attentional biases in a more ecologically valid manner. These research findings and suggestions not only deepen our understanding of fear generalization mechanisms in social anxiety but also provide new directions for clinical intervention. In particular, understanding the modulatory role of evaluative valence may help develop more targeted treatment strategies, such as improved versions of Clark and Wells (1995) cognitive restructuring techniques, to better help socially anxious individuals cope with various social evaluation situations.

## 5 Conclusion

This study employed SSVEP technology to investigate the neural characteristics of early visual processing in social anxiety and the modulatory role of evaluation valence. The results indicate two key findings. First, individuals with high social anxiety exhibit significant fear over-generalization and heightened visual cortical activation, reflecting a sustained hypervigilance to social cues. Second, and more critically, the study suggests a key neural deficit: unlike their low-anxiety peers, high-anxiety individuals fail to use positive evaluative feedback to down-regulate this hypervigilance. This research contributes to cognitive models of social anxiety by proposing a shift in focus from a general hypersensitivity to threat toward a specific impairment in the neural processing of positive social information. These findings offer new directions for clinical intervention, suggesting that treatment could target the restoration of the brain’s adaptive response to positive social evaluation.

## Data Availability

The raw data supporting the conclusions of this article will be made available by the authors, without undue reservation.
